# Hetero-integration enables fast switching time-of-flight sensors for light detection and ranging

**DOI:** 10.1038/s41598-020-59677-x

**Published:** 2020-02-17

**Authors:** Minseong Park, Yongmin Baek, Mesgana Dinare, Doeon Lee, Kyung-Ho Park, Jungho Ahn, Dahee Kim, Joseff Medina, Won-Jin Choi, Sihwan Kim, Changjie Zhou, Junseok Heo, Kyusang Lee

**Affiliations:** 1 Department of Electrical and Computer Engineering, University of Virginia, Charlottesville, VA 22904 USA; 2 Devices Technology Division, Korea Advanced Nano Fab Center (KANC), Suwon, 16229 South Korea; 3 Department of Electrical and Computer Engineering, Ajou University, Suwon, 16499 South Korea; 4 Department of Mechanical and Aerospace engineering, University of Virginia, Charlottesville, VA 22904 USA; 5 RayIR Corporation, LTD, 156 Gwanggyo-ro, Yeongtong-gu, Suwon 16506 South Korea; 6 Department of Science, Jimei university, Jimei District, Xiamen, Fujian, 361021 China; 7 Department of Material Science and Engineering, University of Virginia, Charlottesville, VA 22904 USA

**Keywords:** Electrical and electronic engineering, Imaging and sensing

## Abstract

The time-of-flight (ToF) principle is a method used to measure distance and construct three-dimensional (3D) images by detecting the time or the phase difference between emitted and back-reflected optical flux. The ToF principle has been employed for various applications including light ranging and detection (LiDAR), machine vision and biomedical engineering; however, bulky system size and slow switching speed have hindered the widespread application of ToF technology. To alleviate these issues, a demonstration of hetero-integration of GaN-based high electron mobility transistors (HEMTs) and GaAs-based vertical cavity surface emitting lasers (VCSELs) on a single platform via a cold-welding method was performed. The hetero-integrated ToF sensors show superior switching performance when compared to silicon-transistor-based systems, miniaturizing size and exhibiting stable ranging and high-resolution depth-imaging. This hetero-integrated system of dissimilar material-based high-performance devices suggests a new pathway towards enabling high-resolution 3D imaging and inspires broader range application of heterogeneously integrated electronics and optoelectronics.

## Introduction

Nature includes abundant examples based on the time-of-flight (ToF) principle^1–5^. Echolocation in mammals and birds calculates the time or phase difference between transmitting and receiving particles or waves in order to detect objects remotely^6^. The ToF principle is generally engineered via optical flux in the sensor to achieve high accuracy and frame-rate^7^. Therefore, ToF-based sensors have been widely employed for ranging and mapping technology, leveraging many advanced three-dimensional (3D) applications in machine vision and the biomedical industry etc^8–15^. However, current ToF building blocks, such as emitters, receivers and drivers, are still necessary to simplify their structural complexity, cost-ineffectiveness and large form-factors, and to enhance their insufficient optical power, speed and sensitivity^15,16^. To alleviate these issues, hetero-integration of GaN-based high electron mobility transistors (HEMTs) and GaAs-based vertical cavity surface emitting lasers (VCSELs) on a single platform via a cold-welding process that provides the potential for high resolution 3D real-world imaging is demonstrated.

GaN-based HEMTs have been promising candidates for high power and high frequency applications due to the advantageous intrinsic material properties of GaN for device applications, including low parasitic capacitance, large breakdown endurance and low on-resistance^17–20^. AlGaN/GaN heterostructures in GaN HEMTs allow the formation of two-dimensional electron gas (2DEG) on the channel with a high density of the electron gas (>1 × 10^13^ cm^−2^) by piezoelectric polarization effects, resulting in high speed operation and low node-to-node junction capacitance^18,21^. Recently, GaN-based HEMTs have been employed to drive high-peak and narrow-pulse optoelectrical device applications, including ToF ranging, 3D imaging and light detection and ranging (LiDAR)^16,18^. These applications require sub-nanosecond acquisitions; therefore, the switching performance of devices is critical. Switching performance is affected by gate resistance, capacitance, mobility and inductance of driving transistors. GaN HEMTs show outstanding performance, with faster switching performance than that of silicon-based metal-oxide semiconductor field effect transistors (MOSFETs).

In addition, high-power emitters are the essential device component for efficient pulse-based optical communication. VCSELs are one of the most reliable and high-performing emitters. This is due to their surface-normal structure, large current densities, directive emission, narrow bandwidth, low divergence angle and low power consumption^22,23^. Although VCSEL chips are available with microscale dimensions, additional fabrication or packaging processes are required to be combined with other electronic/optoelectronic components. Monolithic fabrication of modules is advantageous for preventing device degradation related to material incompatibility and processes reliability^24^. Despite this, straightforward and inexpensive processes are still challenging.

Heterogeneous integration provides the potential for system-level applications without the issues of incompatibilities stemming from intrinsic material properties, complex spatial layout and geometric limitation^25–29^. Here, we fabricated a hetero-integrated ToF sensor was fabricated by combining depletion-mode AlGaN/GaN HEMTs and VCSELs via the cold-welding process. The simple cold-welding process enables quick and low-temperature bonding for microscopic structures^30^. The hetero-integrated ToF sensor exhibits the superior switching performance for advanced 3D optical imaging systems. The novel ToF device is expected to shed light on exhibiting high-resolution 3D imaging as well as enhance the structural degree of freedom.

## Results

### Characterization of GaN HEMTs and GaAs VCSELs

**Figure 1:**
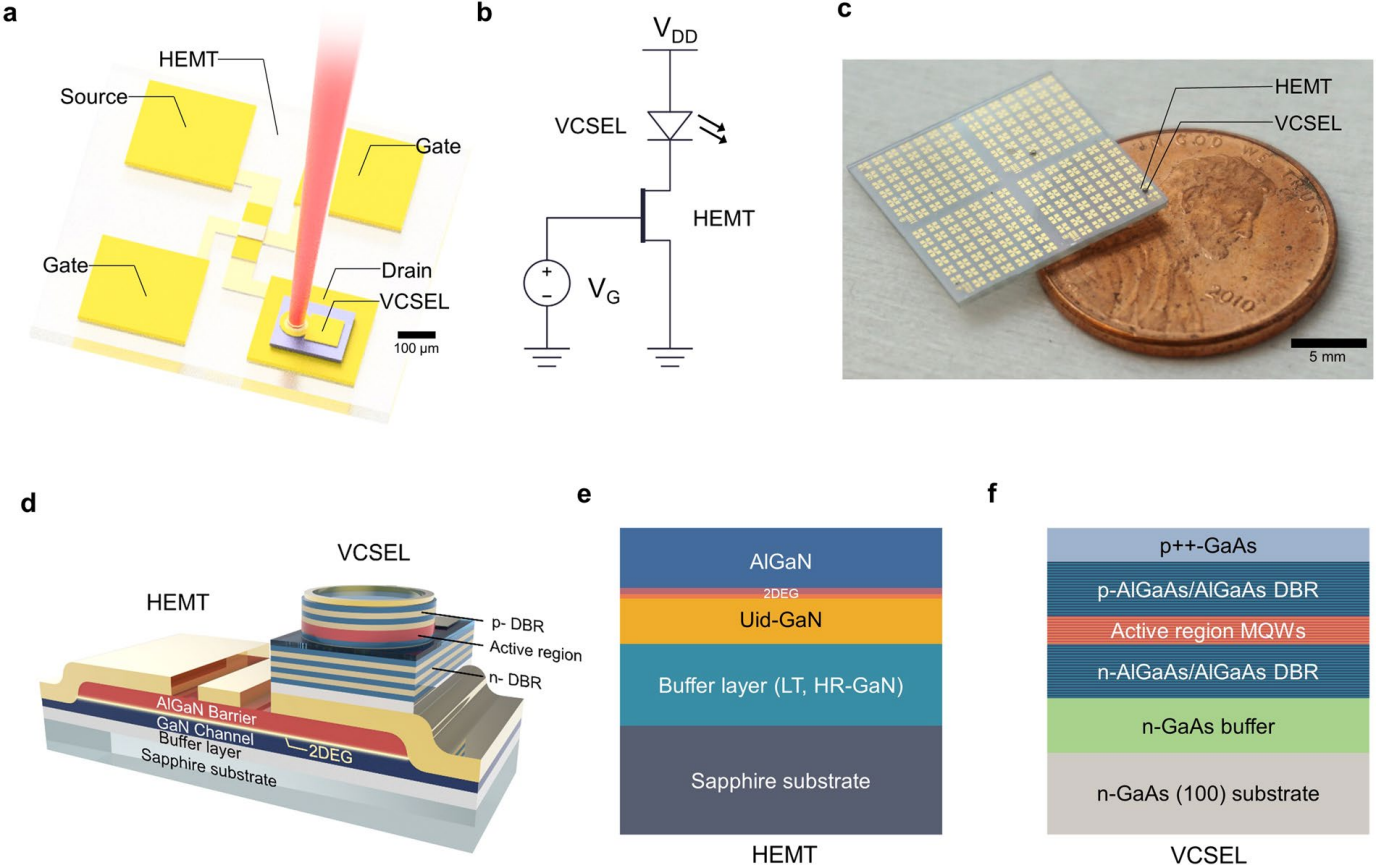
Structures of the hetero-integrated devices. (**a)** 3D schematic illustration of the devices. The VCSEL is integrated into the drain region of the HEMT. Scale bar: 100 µm. (**b)** A circuit diagram and **c** photograph of the hetero-integrated device on a penny. Scale bar: 5 mm. (**d)** A 3D structure of the device (not scaled). (**e)** A cross-section of the GaN-HEMTs. A low temperature (LT) and high resistance (HR) GaN buffer layer are sandwiched between the substrate and the unintentionally doped (Uid) GaN layer. (**f)** A cross-section of GaAs**-**VCSELs. Multiple-quantum-wells (MQWs) are sandwiched between doped distributed-Bragg-reflector (DBR) layers.

Prior to hetero-integration, the electrical and optical characteristics of GaN-based HEMTs and GaAs-based VCSELs was investigated. Figure 1a shows a schematic illustration of the hetero-integrated device. The VCSEL chip is integrated on the drain side of the GaN HEMT to prevent fluctuations related to turn-on stages from affecting the source voltage of the GaN HEMT, as shown in Fig. 1b. Figure 1c shows a photograph of the hetero-integrated device. The cross-sectional structure of the device is shown in Fig. 1d. The 2DEG layer in the GaN-based HEMT where carriers are accumulated enhances the electrical conductivity of the device, thus enabling high current density. Figure 1e,f show an epitaxial cross-section of the GaN-based HEMT and VCSEL. Each component is grown on a different substrate (sapphire and n-GaAs substrate). After the independent fabrication process of each component, the VCSEL chip and GaN-based HEMT are hetero-integrated through cold-welding. (For more detailed fabrication process and hetero-integration, see Methods).

**Figure 2:**
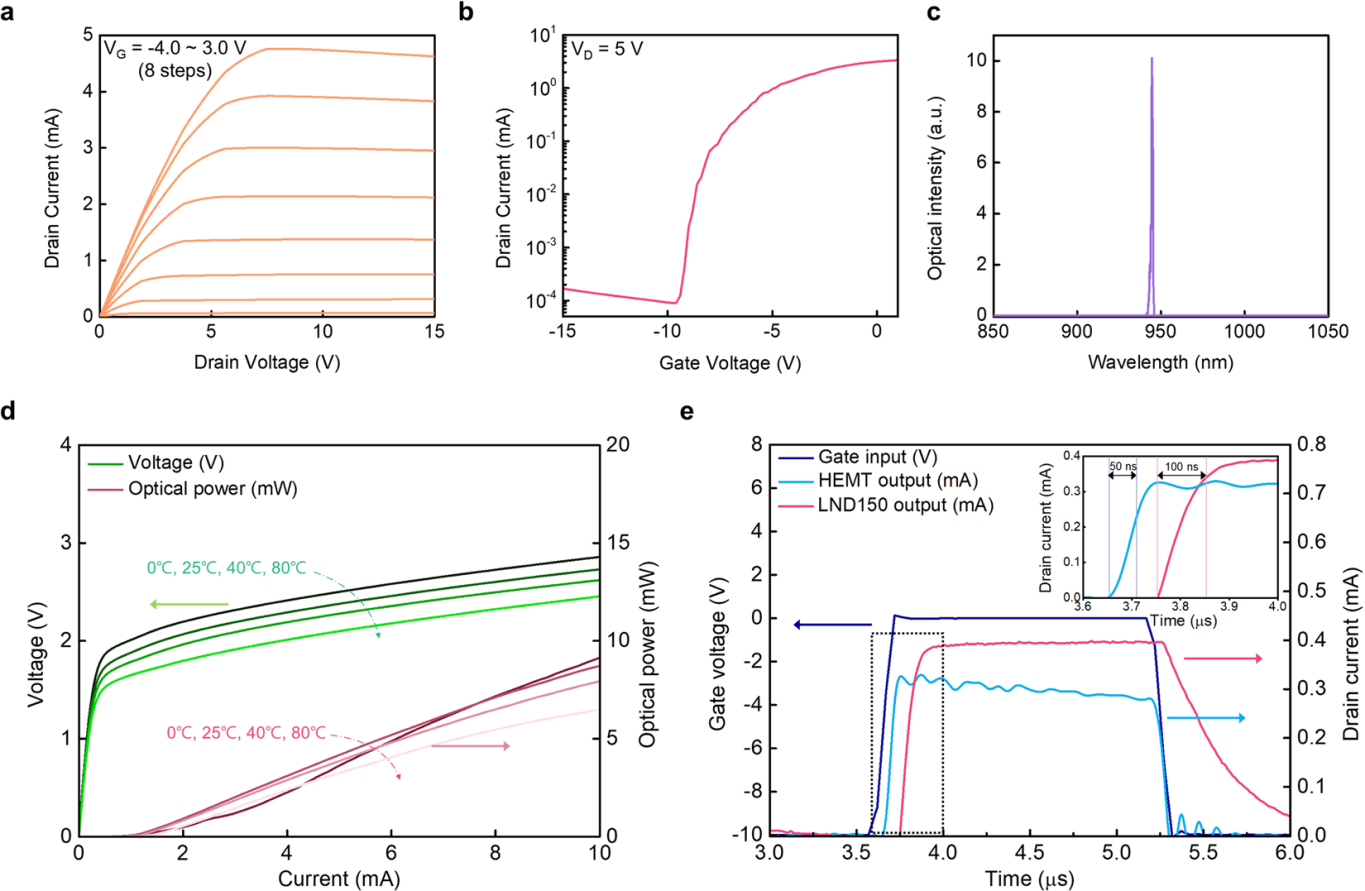
Electrical and optical characteristics of GaN HEMTs and VCSELs. (**a)** Output characteristics of the AlGaN/GaN HEMTs under various gate voltages. The gate voltages are sequentially applied from −4.0 V to 3.0 V with 8 steps (starting from the very bottom orange curve). (**b)** A transfer curve of the HEMTs with the drain voltage (5 V). The threshold voltage is −7 V. (**c)** Spectroscopy of the emitting light from VCSELs. The peak wavelength is 945 nm. (**d)** L-I-V characteristics of the VCSELs at four different temperatures (0 °C, 25 °C, 40 °C, and 80 °C respectively, starting from the darkest curve). The green lines are voltage (left y-axis), and the red lines are optical power (right y-axis). The optical power is measured at 8 mA, and the turn-on voltage ranges from 1.5 V to 1.9 V. **e** Rise and fall time response of the AlGaN/GaN HEMTs and Si-MOSFETs (LND 150). The navy line is gate voltage (left y-axis), and the red and blue lines are drain current (right y-axis). Inset: close-up of the black-dotted area to compare rising responses at the rising interval (3.6 µs~4.0 µs). The two rise times are measured from 90% of pulse-on voltage and 10% of pulse-off voltage. (For more details, see Methods).

Figure 2a shows the current-voltage (I-V) characteristics of the AlGaN/GaN HEMT. The current at the saturation regions slightly decreases at the high gate voltage (1.0 V~3.0 V) and the high drain voltage (>10 V), due to the self-heating effects^31^. The threshold voltage is −7 V, as shown in Fig. 2b. Based on these I-V characteristics of GaN-based HEMT, the stable operating regions, where gate voltage ranges from −10 V to 2.0 V and drain voltage is below 10 V, are then adopted as pulse levels for ToF function. The fabricated VCSEL shows 20 degrees of beam divergence without a focusing lens, and a monochromatic spectrum wavelength of 945 nm, as shown in Fig. 2c. The narrow wavelength spectra emission contributes to higher signal-to-noise ratio (SNR). Furthermore, the scalability and surface normal emission of the VCSELs are ideal for structural flexibility of ToF systems. The light-current-voltage (L-I-V) characteristics of the VCSELs are shown in Fig. 2d. The threshold currents are nearly constant at various temperatures. Thermal effects slightly diminish optical power as the operating temperature increases. The fabricated phase-based ToF module can operate reliably up to 85 °C, which is similar to the operating temperature of commercialized ToF modules^32^. The current level is determined by device endurance and power conversion efficiency; thus, less than 8 mA current is applied to the hetero-integrated device. Figure 2e confirms advantageous fast switching of the fabricated GaN HEMTs. The rise time of the HEMTs is 50 ns, which is over twice as fast as that of the commercialized Si-MOSFETs (Microchip Technology, LND 150). Furthermore, the fall time of HEMTs is remarkably faster than that of MOSFETs as well. The superior rise and fall times of the fabricated GaN-based HEMT are comparable to those of commercial GaN-based HEMTs^33^. The difference in turn-on delay times of both HEMTs and Si-MOSFETs originates from the difference in threshold voltages; GaN HEMTs with −7 V and Si-MOSFETs with −1.5 V. The short rise and fall times support the performance of GaN HEMTs as fast switching devices to drive the current for a ToF sensor.

### Hetero-integration of GaN HEMTs and GaAs VCSELs

**Figure 3:**
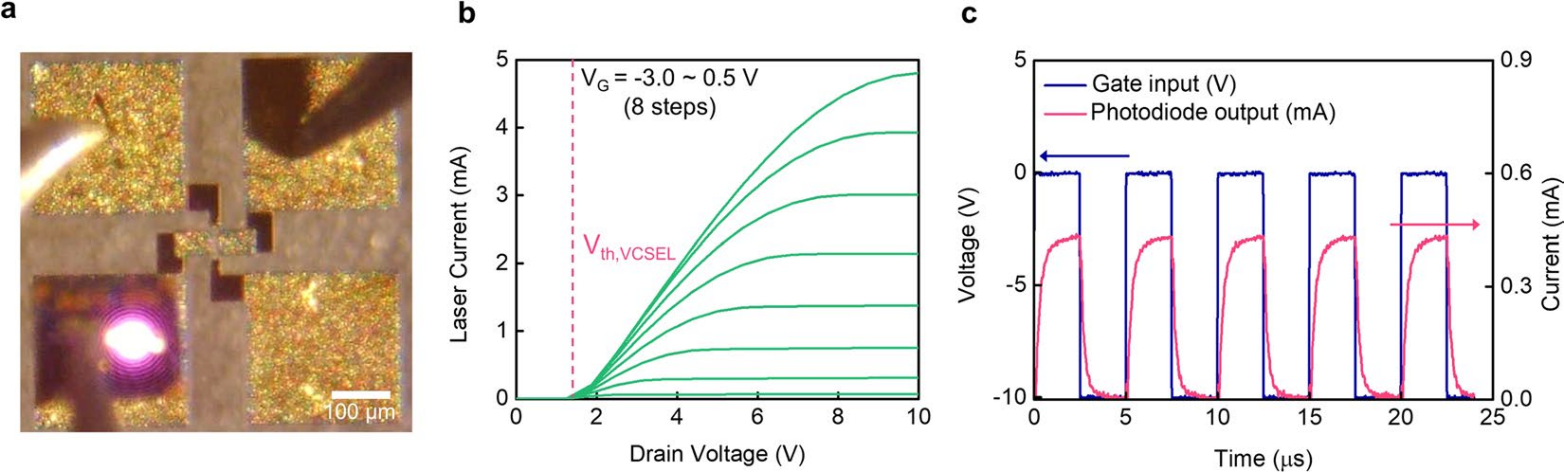
Characteristics of the hetero-integrated devices. (**a)** A microscopy image of the device on probe stations. The scale bar is 100 µm. The invisible infrared emission is converted to a purple beam. (**b)** I-V characteristics of the heterogeneously integrated device under various gate voltages. Eight-stepped gate voltages are applied sequentially from −3.0 V to 0.5 V (starting from the very bottom green curve). Red dot line shows the turn-on voltage of the VCSELs. (**c)** Photodiode responses from the reflected beam. The navy line is voltage (left y-axis), and the red line is current (right y-axis). The pulse width is 2.5 µs, and the repetition rate is 200 kHz. The VCSEL is activated by gate pulses of the hetero-integrated device. The navy line is gate input (V) and the pink line is photodiode output (µA).

Hetero-integration plays a key role in systemizing nanosecond-switching and miniaturizing integrated devices^30,34^. Compared to conventional wire bonding, direct interfaces via hetero-integration minimize redundant physical connections to relax parasitic inductance and capacitance. This results in an accurate ToF calculation from the speed of light and enhanced power efficiency. A cold-welding method was used to heterogeneously integrate the GaN HEMTs and GaAs VCSELs. This simplified the fabrication process and minimized the damage and degradation of device performance due to high temperatures (see Methods)^35,36^. Electro-optical responses of the hetero-integrated devices for ToF are analyzed in Fig. 3. Since the emission wavelength of the VCSELs is in the near-infrared range (peak at 945 nm), image sensors recognized the emission in a conventional microscope, as shown in Fig. 3a. Figure 3b shows the I-V characteristics of the hetero-integrated devices. Above the threshold voltage (1.5 V), the current followed the I-V behavior of the HEMTs. Detailed measurement is described in Methods. The voltage pulse was then applied in order to characterize optical response, as shown in Fig. 3c. The emitted optical beam from VCSEL was reflected from a scattering object, and the photocurrent of the photodiode was generated by the reflected near-infrared beam. The photodiode was activated in accordance with the timing of the turn-on gate pulse. The VCSEL was activated at a fast pulse repetition rate, up to 500 kHz. Further improvement can be possibly achieved if the peripheral circuits and measurement setup are optimized (See Supplementary Fig. 3).

### Hetero-integrated time-of-flight devices

**Figure 4:**
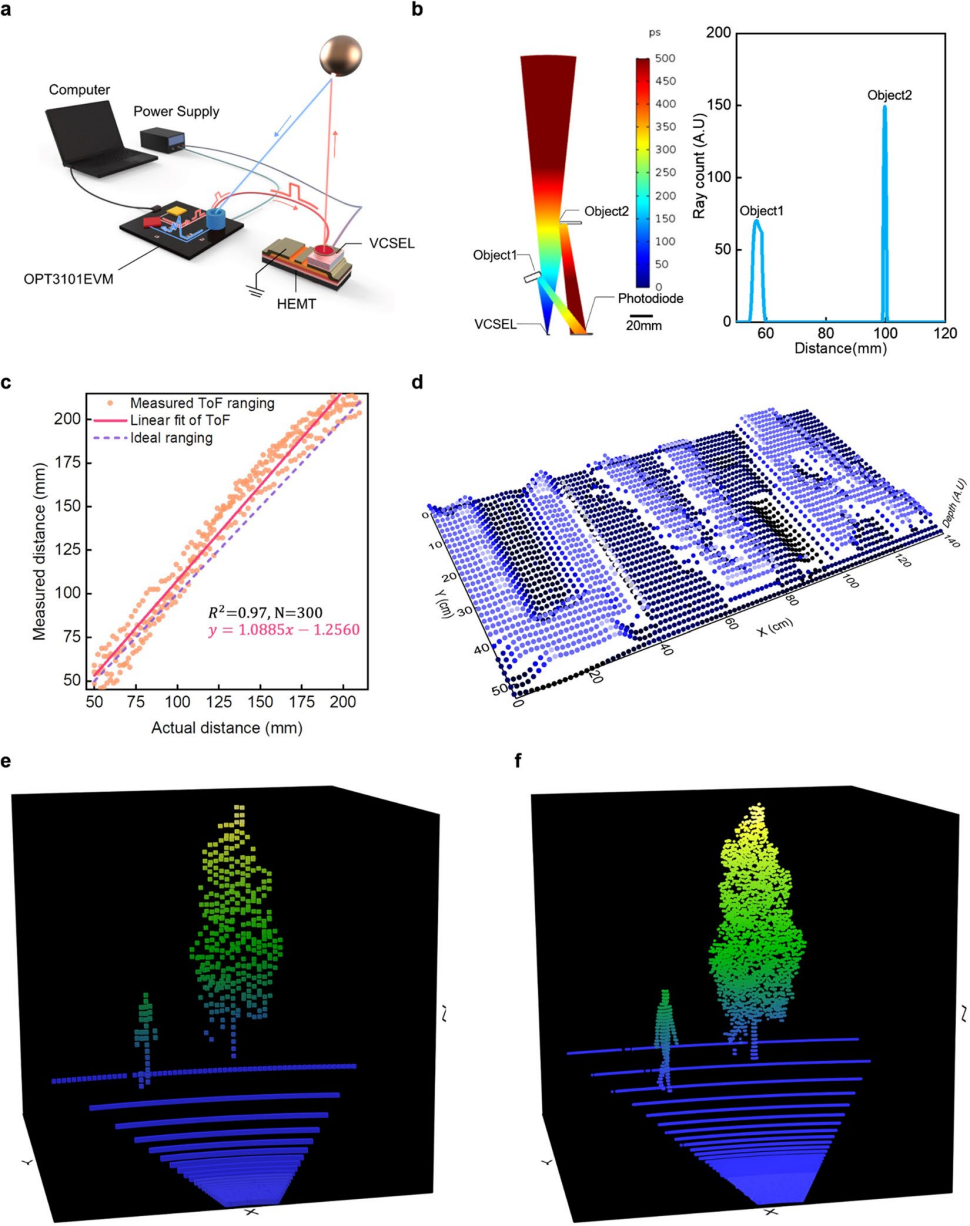
ToF ranging and imaging via the hetero-integrated devices. (**a)** Experimental setup of ToF ranging. The object moves along normal to the surface of the hetero-integrated device. (**b)** A geometrical ray tracing simulation of ToF sensors with results. The scale bar is 20 mm. The color bar shows a picosecond-scale of each point of the ray (left). Blue line: ToF response of each object (right). (**c)** Linear results of ToF ranging. The minimum distance is 50 mm, and the maximum distance is 210 mm. 300 measurements are shown as orange dots, and the ideal ranging line is shown as a black-dotted line (Actual distance = Measured distance). The R-square linear fitting parameter is 0.97. (**d)** 3D ToF imaging of ‘UVA’ letters. The z-axis (depth) is an arbitrary unit. 32 × 32 grids per each letter are adopted. Simulations of ToF imaging using **e** commercialized ToF, and (**f)** this work. The original image is one man with a tree (For more details, see Methods). Colormaps are indicated as heights (blue: 0 cm and yellow: 800 cm).

The ToF experimental setup that allowed measurement of a real-time depth curve is illustrated in Fig. 4a. Detailed information of the setup is demonstrated in Methods and Supplementary Fig. S1. Prior to the experiment, ToF function was geometrically simulated using COMSOL multiphysics software, as shown in Fig. 4b (For more information, see Methods). The object located normal to the emitter (Object 2) allowed confined optical responses compared to the object located at an off angle from the sensor. Therefore, for the calibration of ToF ranging, the object moved along normal to the surface of the emitter to output multiple distances between the object and the hetero-integrated ToF. The characterization of ToF measurement is shown in Fig. 4c. The device exhibited consistent trends with the transition of the distance between the object and the VCSEL. The distribution of the 300 measurements was interpreted as a linear regression model with a reliable R-square value (0.97). The device also showed stable ranging for a long-term single-target (See Supplementary Fig. S2). Furthermore, the hetero-integrated device enabled ToF-based 3D imaging, as shown in Fig. 4d. ‘UVA’ letters were used for this 3D depth imaging. The edges of each letter are recognizable, which supports the possibility of using a demonstrated ToF sensor for object and material recognition applications^37–40^. The fast switching performance of the hetero-integrated devices also elicits great potential for a spacious mapping application, such as LiDAR, where high-resolution can be realized by fast pulse repetition rate. The simulated results using the characterization of the fabricated ToF sensor show high-resolution 3D imaging, as compared to the conventional Si-MOSFET-based LiDAR system, displayed in Fig. 3e,f (see Method)^41^. Compared to the conventional LiDAR system, the superior fast-switching and high-power performance of the hetero-integrated device exhibited higher pulse repetition rate and SNR. Therefore, more compact 3D point data is implemented via the hetero-integrated device. These results support the prospect of using compact hetero-integrated devices to realize real-time 3D mapping as well.

## Discussion

Direct ToF ranging is calculated as $d=\frac{1}{2}c\cdot {\tau }_{ToF}$, where *d* is the distance between a sensor and object, *c* is the speed of light, and ${\tau }_{ToF}$ is the time difference between transmitter and receiver signals. The ${\tau }_{ToF}$ can be derived from time-to-digital converters (TDCs), but can also be calculated by the phase difference between the transmitter and receiver signals as $\varphi =2{\rm{\pi }}f{\tau }_{ToF}$ and $d=\frac{c}{4{\rm{\pi }}f}\varphi$, where $f$ is modulation frequency, and $\varphi$ is a phase difference between illumination and reflection. The phase difference is calculated from the intensity of received signal at four different points such that $\varphi =arctan(\frac{{A}_{1}-{A}_{3}}{{A}_{2}-{A}_{4}})$, where A_1_, A_2_, A_3_ and A_4_ are the measurements at four different phases (0 deg, 90 deg, 180 deg and 270 deg each). The phase-shift-based ToF model was employed in this study due to its high accuracy in the range of millimeters with high surface reflectivity. The noise at the edge of the square waves was mitigated by damping the parasitic circuits to relax the resonance behavior. The mismatch between the linear fit and the ideal line was possibly caused by an inertial fluctuation of the setup and a lack of further precise calibration without consideration of humidity and ambient light conditions. These factors are mostly related to the SNR and elicit measurement deviation as ${{\rm{\sigma }}}_{distance}=\frac{d}{SNR}$. Normally, one millimeter accuracy corresponds to 6.6 picoseconds pulses, which is challenging for a conventional silicon-based photodiode^7^. In this experiment, the range was 50 mm to 210 mm in order to maintain the minimum distance between the microprobes and object, and the maximum distance was limited by the optical power of the emitter and detectivity of the photodetector. Therefore, further ranging is possible by maximizing the SNR using a high optical power emitter with a high sensitivity receiver. Sub-watt optical power of the VCSELs can be easily achieved by employing multiple VCSEL-based emitters^42–44^. Moreover, the VCSEL beamwidth is small and directional. Thus, combination with additional beam controllers, such as rotary motors^16^, micromirrors^45,46^, optical phased array (OPA)^47–49^, and liquid crystal (LC) based beam steerers^50^, is expected to pave the way for future angle-dependent ranging and high-resolution 3D imaging.

In summary, HEMTs were fabricated and heterogeneously integrated with VCSELs for ToF ranging via cold-welding. The superior rise and fall time of the GaN HEMTs over conventional Si-MOSFETs enables fast-switching devices. The as-fabricated HEMTs and VCSELs operate in comparable voltage (gate voltage below 2 V and drain voltage below 10 V) and current levels (8 mA) for a ToF function. The fabricated VCSELs display narrow wavelength spectra and beam emission, thus making VCSELs ideal for ToF systems. The simple hetero-integration allows further fast-switching performance of the devices by minimizing device degradation and parasitic connection. This high-speed switching performance is then utilized to exhibit accurate ToF-based distance ranging and imaging and potential to facilitate high-resolution 3D imaging. We believe that the hetero-integrated device is expected to open the way for alternatives to current formalized ToF sensors, boosting the emergence of newly outlined 3D sensors via their high degree of scalability and compatibility.

## Methods

### Device fabrication

The cross-section structure of each AlGaN/GaN HEMTs and GaAs VCSELs are illustrated in Fig. 1e,f. The heterostructure of the AlGaN/GaN HEMT was grown on a sapphire substrate by metal organic chemical vapor deposition (MOCVD). The low-temperature (LT) and high resistance (HR) layer were grown as a buffer layer for the high-quality growth of the epitaxial GaN HEMT structure. For device fabrication, after the mesa etching, ohmic contacts were metallized and improved by annealing using rapid thermal annealing (RTA). Finally, gold pads for source, drain, and gate were deposited using e-beam evaporation. The VCSEL structure was grown by MOCVD on a GaAs substrate. The epilayer structure is composed of three InGaAs–AlGaAs multiple-quantum-wells (MQWs) sandwiched between a 38-pair n-type and 21-pair p-type Al_0.90_Ga_0.10_As–Al_0.05_Ga_0.95_As distributed-Bragg-reflector (DBR) layers with an p-type Al_0.98_Ga_0.02_As layer (30 nm thickness) above the MQWs for oxidation. An oxidation technique was used to define a circular current-confined area of 10 µm in diameter. The 10-µm aperture size has been employed to maintain the consistency of the fabrication and to achieve the optimum number of cavity modes and gain for our applications. On the backside of the GaAs substrate, gold film was deposited by e-beam evaporation, which serves as a bottom electrode. Subsequently, a VCSEL chip was integrated on a HEMT using a thermally-assisted cold-welding method^51^. 200 MPa force is applied to the pre-cleaned surface between the two gold films at 270 °C. To apply uniform force over the interfaced area, a Teflon film is inserted between the sample and the pressing head.

### Electrical characterization

Electrical performance of the hetero-integrated devices was characterized by a KEYSIGHT B1500A Semiconductor Device Analyzer equipped with a waveform generator/fast measurement unit, a pulse generator (KEYSIGHT 33600A Series), and Digilent Analog Discovery 2. To measure pulse repetition rate (PRF) and photodiode current, an oscilloscope (KEYSIGHT DSO-X 3024T), and current amplifier (Edmund 59–179) were employed to minimize external noise. A continuous measurement mode was adopted for the I-V characteristics. An additional power supplier (GS-1325-ND) was used for ToF ranging and imaging. With a Si-based counterpart reference device for comparison, LND 150 commercially available Si-MOSFETs and SFH2505 photodiodes (OSRAM Opto Semiconductors) were adopted.

### Ray tracing simulations

COMSOL Multiphysics software was used for the ray tracing simulation. To implement the simulation, the two-dimensional model with geometrical optics (GOP), and ray tracing study was adopted. The refractive index of the objects was 3.6. In the GOP section, the maximum number of secondary rays were 500, and 940 nm was employed for the ray properties. The cone angle of the emitted beam from VCSEL was 10 degrees. For the surface property of the object for simulation, objects with the specular reflection surface conditions were used for convenience. A ray detector was added to count photons passing through the detecting area.

### Time-of-flight implementation

Phase shift-based ToF was employed for this study. To record the exact phase difference from both transmitter and receiver signal, OPT3101 (Texas Instruments) analog front-end (AFE) was used. The transmitter square-shaped waves are based on continuous-wave modulation, which was then used to calculate the phase difference between transmitted and received signal. The calibration is performed to eliminate the crosstalk caused by the background signal for precise measurement. To calibrate the module, the software development kit (SDK) code was run at various temperatures to measure both crosstalk and phase. Then, the phase at various ambient light brightness was measured; the values gained and extracted from the calibration were used to the correct SDK method which relates these values to register writes.

### 3D ToF imaging simulations

Heidelberg LiDAR operations simulator (Helios, GIScience) software package was utilized to generate 3D point cloud data. The optical flux is collimated, the optical power is 4 W, and the wavelength of the simulation is the same wavelength used in this work (945 nm). Scanning angle is 50 degrees, the pulse width is 30 ns, and the scanning frequency is 500 Hz. With this simulation, the resolution of commercialized LiDAR was compared to the work done in this study; thus, the horizontal scanning speed (${v}_{h}$), vertical scanning speed (${v}_{v}$), and total scanning time (${\rm{t}}$) are the same. Since the number of ranged 3D points are proportional to ${v}_{h}{v}_{v}t\times PRF$, PRF determines the lateral resolution of imaging, and maximum ranging distance. The ranging resolution is determined by response time of each LiDAR components, such as an emitter, receiver and peripheral circuits. In the case of the simulation for commercialized LiDAR, 40 kHz PRF is adopted. 500 kHz PRF is adopted for this work, which is the identical frequency measured using the fabricated hetero-integrated ToF sensor (See Supplementary Fig. S3).

## Supplementary information

Supplementary information

## Data Availability

The data related to the figures and other findings of this study are available from the corresponding author upon reasonable request.
