# Supplementary material for: Hetero-integration enables fast switching time-of-flight sensors for light detection and ranging
Source: Sci Rep. 2020 Feb 17;10:2764. doi: 10.1038/s41598-020-59677-x (PMC7026395; doi:10.1038/s41598-020-59677-x)
Supplement: Supplementary file 1 — Supplementary information [file 41598_2020_59677_MOESM1_ESM.pdf]

Supplementary information

Hetero-integration enables fast switching time-of-flight sensors for light detection and ranging

Minseong Park, Yongmin Baek, Mesgana Dinare, Doeon Lee, Kyung-Ho Park, Jungho Ahn, Dahee Kim, Joseff Medina, Won-Jin Choi, Sihwan Kim, Changjie Zhou, Junseok Heo & Kyusang Lee

Supplementary Figures

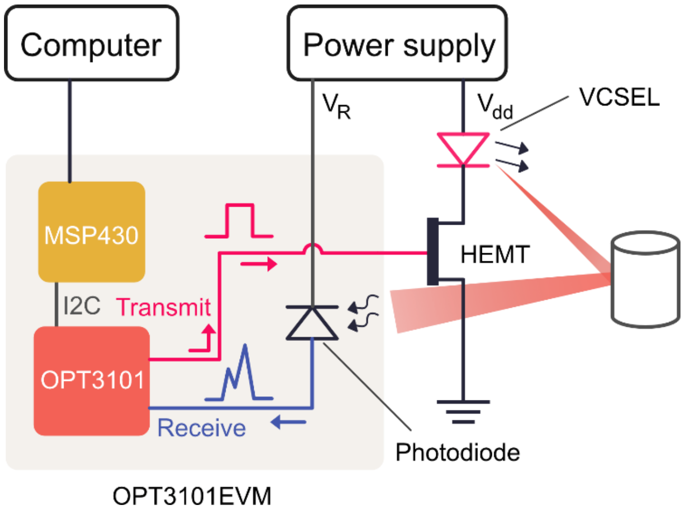

Supplementary Figure. 1 Experimental setup of time-of-flight (ToF) ranging and imaging by hetero-integrated devices. Since a time-scale of the ToF is from picosecond to sub-nanosecond, accurate signal acquisition is indispensable. Thus, the microcontroller (MSP430) generates and synchronize timing to transmit and receive signals. The computer supplies power to MSP40 and OPT3101. The power supply generates two distinct voltages to the detector and the hetero-integrated device, respectively. Calibration is carried out in this setup to include all ambient effects out of the OPT3101EVM.

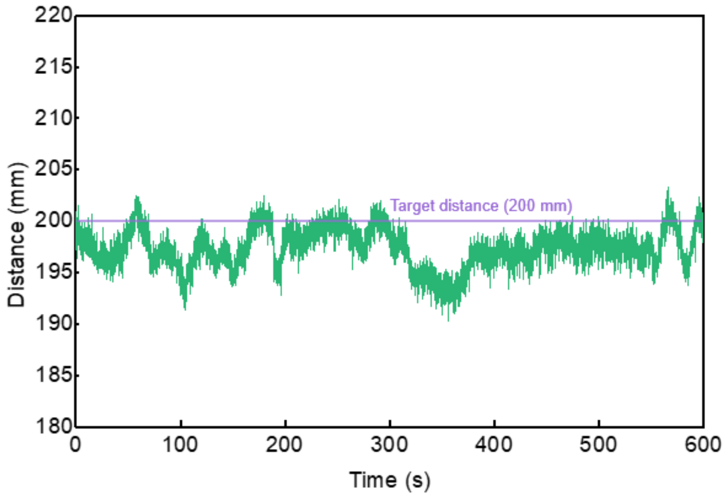

Supplementary Figure. 2 Long-term ToF ranging for a single target. The object is fixed at 200 mm to the VCSEL (purple line). 19,800 samples are measured for 10 minutes. (green line)

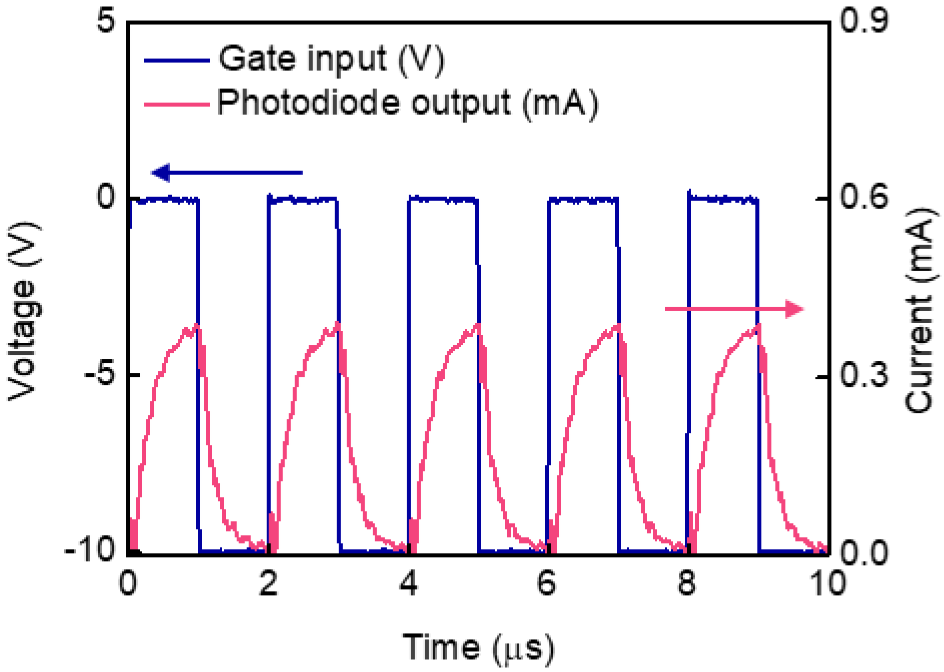

Supplementary Figure. 3 Photodiode responses from the reflected beam. The navy line is voltage (left y-axis), and the red line is current (right y-axis). The pulse width is 1 μs, and repetition rate is 500 kHz. The navy line is gate input (V) and the pink line is photodiode output (μA).
